# Additive Manufacturing of β-Tricalcium Phosphate Components via Fused Deposition of Ceramics (FDC)

**DOI:** 10.3390/ma14010156

**Published:** 2020-12-31

**Authors:** Steffen Esslinger, Axel Grebhardt, Jonas Jaeger, Frank Kern, Andreas Killinger, Christian Bonten, Rainer Gadow

**Affiliations:** 1 Institute for Manufacturing Technologies of Ceramic Components and Composites, University of Stuttgart, Allmandring 7b, 70569 Stuttgart, Germany; frank.kern@ifkb.uni-stuttgart.de (F.K.); andreas.killinger@ifkb.uni-stuttgart.de (A.K.); rainer.gadow@ifkb.uni-stuttgart.de (R.G.); 2 Institut fuer Kunststofftechnik, University of Stuttgart, Pfaffenwaldring 32, 70569 Stuttgart, Germany; axel.grebhardt@ikt.uni-stuttgart.de (A.G.); christian.bonten@ikt.uni-stuttgart.de (C.B.); 3 Hahn-Schickard-Gesellschaft fuer angewandte Forschung e.V., Allmandring 9b, 70569 Stuttgart, Germany; jonas.jaeger@hahn-schickard.de

**Keywords:** additive manufacturing, fused deposition of ceramics (FDC), filament extrusion, calcium phosphate, scaffold, poly(lactic acid), poly(ethylene glycol)

## Abstract

Bone defects introduced by accidents or diseases are very painful for the patient and their treatment leads to high expenses for the healthcare systems. When a bone defect reaches a critical size, the body is not able to restore this defect by itself. In this case a bone graft is required, either an autologous one taken from the patient or an artificial one made of a bioceramic material such as calcium phosphate. In this study β-tricalcium phosphate (β-TCP) was dispersed in a polymer matrix containing poly(lactic acid) (PLA) and poly(ethylene glycole) (PEG). These compounds were extruded to filaments, which were used for 3D printing of cylindrical scaffolds via Fused Deposition of Ceramics (FDC) technique. After shaping, the printed parts were debindered and sintered. The components combined macro- and micropores with a pore size of 1 mm and 0.01 mm, respectively, which are considered beneficial for bone healing. The compressive strength of sintered cylindrical scaffolds exceeded 72 MPa at an open porosity of 35%. The FDC approach seems promising for manufacturing patient specific bioceramic bone grafts.

## 1. Introduction

Bone loss generally has a significant impact on patients’ quality of life. As life expectancy increases and the world population ages, there is also an increase in musculoskeletal pathologies such as fractures, osteoporosis, and osteoarthritis. While smaller bone defects can be repaired by the body itself, fractures exceeding a critical size require medical treatment. In this case often a bone graft is needed [1]. Besides blood bone is the most frequently transplanted tissue [2]. From a medical point of view, autologous bone grafts are still considered as the “gold standard” [3,4]. This means that the donor and recipient are identical. The main advantage of autologous bone is its high compatibility. However, this approach also has disadvantages, for example due to the low availability of autologous bone, the additional surgeries that are painful for the patient and the risk of removal morbidity. Allografts (from living human donors or corpses) or xenografts (from animals) are more widely available but carry the risk of pathogen transmission and implant rejection by the recipient’s body [5].

Scaffolds made of plastic, metal, ceramic, or composite materials are another source of supply for bone grafts. Properties of these artificial bone substitutes have to meet a complex set of requirements. The most important property is the biocompatibility of the implants, which means that they must not have any damaging effect on the surrounding tissue or the organism. Possible damage to healthy bones may arise from the release of acids from degrading polymers. Metal constituents of grafts may cause allergic reactions. Certain groups of materials such as bioactive ceramics are able to accelerate the regeneration of the bone, to stimulate cell proliferation and, at the same time, to degrade and transform themselves into the body’s own material. The advantage of these materials is that no further surgery for the potential removal of parts of the implant is necessary for the patient or no artificial material has to remain in the body, as is the case, for example, with metallic screws, plates or artificial metal, polymer, or ceramic joints [6,7].

Calcium phosphate ceramics (CaP) are among the most widely used and effective synthetic bone substitute materials. For example, β-tricalcium phosphate (β-TCP) is osteoconductive and is integrated into the bone without a disruptive layer of connective tissue [8,9]. This property, combined with its cell-mediated resorption, enables the complete regeneration of bone defects. Pores and especially micropores (0.1 to 10 µm) promote bone ingrowth and can also give the β-TCP osteoinductive properties [8,10].

Certain restrictions must also be considered with regard to the mechanical properties. According to Wolff’s law of bone transformation, a permanent remodeling of the bone takes place within the body depending on the applied load [11]. Hence the bone implant should have similar properties in terms of porosity, strength, and stiffness as the piece of bone to be replaced. If the strength of the implant is too low, there is a risk that the component will fail after implantation. If the strength and stiffness of the implant are too high, however, the surrounding bone will degrade. This process is called “stress shielding” and, like component failure, should be avoided in any case [12]. The compressive strength of spongy cancellous bones is between 2–20 MPa, depending on the literature. In terms of porosity, the cancellous bone exhibits values between 50–90%, which explains the limited load bearing capacity. At the same time, however, the bone becomes light and the pores allow the bone to be supplied with nutrients and metabolic products to be removed [13,14]. In principle, the authors of scientific works agree that a scaffold should have the highest possible porosity. However, there are competing views as to the optimal size of the macropores. While pore sizes between 100–500 µm have been postulated as the optimum in the past decades [15,16], recent publications indicate the beneficial effect of even larger pores in the range between 0.7–1.2 mm [17,18]. The majority of studies on CaP scaffolds focus on bone growth in the macropores (>100 µm), where bone structures such as osteons and trabeculae can form. However, more and more studies show that micropores (<100 µm) not only play an important role in improving bone growth in the macropores, but also in providing additional space for bone growth—they therefore improve and accelerate bone healing [10]. The production of such filigree bioceramic scaffolds with defined macro- and micropores can take place in various ways, such as foaming processes or by means of additive manufacturing. Within the group of additive manufacturing technologies, further sub-processes can be distinguished, such as binder jetting, stereolithography, or Fused Filament Fabrication (FFF), all of which have certain advantages and disadvantages [19,20].

In previous studies we have used the binder jetting technique and the iSFF approach which combined the FFF and the ceramic slip casting process to manufacture bioceramic scaffolds with similar geometries like in this study [21,22,23]. The β-TCP scaffolds made via binder jetting had a porosity of more than 63%, while their compressive strength of 0.6 MPa was extremely low. With the iSFF approach the compressive strength could be increased up to 16 MPa at a corresponding porosity of only 16%.

The present study aims at manufacturing β-TCP scaffolds by the Fused Deposition of Ceramics process. For this shaping technique the bioceramic powder is dispersed in organic components and extruded into filaments, which can be processed in a subsequent step using an FFF printer. The organic components are then debindered in several stages. Finally, the components are sintered at high temperature and mechanically characterized. The issues addressed are:

- to investigate which pore distribution and compressive strength can be achieved
- to find out how this approach contrasts to other 3D-printing techniques
- if the components produced are basically conceivable as cancellous bone substitutes.

## 2. Materials and Methods

### 2.1. Filament Extrusion

#### 2.1.1. Ceramic Powder Preparation

The β-TCP ceramic powders used in this study were received by Chemische Fabrik Budenheim (Budenheim, Germany). The measurement of the particle size distribution was carried out with a laser granulometer Mastersizer 3000 (Malvern Panalytical, Malvern, United Kingdom). The ceramic powder was added into a water-based measuring cell without using ultrasound until the obscuration level reached 5%. The assumed refractive index for the calculation of the particle sizes according to the Mie model was 1.627. The medium particle size d_50_ of the raw material was 6.1 µm, the d_90_ was 14.1 µm. For the milling of the β-TCP the powder was dispersed in distilled water, adding 0.5 wt.% dispersant DOLAPIX CE64 (Zschimmer & Schwarz, Lahnstein, Germany). The milling was carried out in an attritor mill with water cooling using 2 mm Y-TZP balls until d_50_ and d_90_ were smaller than 0.9 µm and 1.8 µm, respectively. After milling for 9–10 h at 500 rpm this particle size was reached, the milling media were removed, and the dispersion medium was allowed to evaporate.

The as received raw powder was mixed with the milled powder in a ratio hereby 70: 30 in order to achieve a bimodal grain size distribution and thereby increase the ceramics content of the filaments.

#### 2.1.2. Organic Components

The organic backbone in the filaments was a poly(lactic acid) (PLA) named Ingeo Biopolymer 4032D (NatureWorks, Blair, NE, USA). PLA was chosen because it is a bio-based material and is a common material in FFF. Three different polymer blends were studied, (see Section 2.1.3), pure PLA and two blends containing different fractions of poly(ethylene glycole) (PEG) named PEG 3000 (Merck Group, Darmstadt, Germany). PEG was selected as a water-soluble component to allow two-stage debinding. The molecular weight of the used PLA was 107,296 g/mol and 3000 g/mol for the PEG. Before compounding the PLA and the PEG were dried in vacuum for 24 h, the temperatures for drying were 50 °C for PLA and 30 °C for PEG, respectively.

#### 2.1.3. Compounding and Filament Extrusion

For the compounding, a twin screw extruder EBVP25 (OMC, Milano, Italy) was used. Three different compounds were produced, see Table 1. The following filament extrusion was carried out by using a single screw extruder 30 × 25D (COLLIN Lab and Pilot Solutions GmbH, Maitenbeth, Germany). The compounds pass through five temperature zones during extrusion. The temperatures during the extrusion of C1, C2, and C3 are documented in Table 2. According to the fact, that PEG has a lower melting point than PLA, the temperature in the different zones was lowered by 10–15 K to increase the viscosity of the extruded filaments. This was necessary to get filaments that can be processed by the Fused Deposition of Ceramics (FDC) printer. The extruded filaments were cooled in a water bath and cut into 400–500 mm rods.

### 2.2. Determination of the Rheological Properties

A rotary rheometer SR-500 (Rheometric Scientific GmbH, München, Germany) was used for the viscosity measurements. The plate spacing was set at 1 mm and the maximum deformation was set at 1%. The compounds C1 to C3 and the pure PLA polymer were examined at a temperature of 180 °C.

### 2.3. Shaping via Fused Deposition of Ceramics

For the printing of the rods a commercially available FFF printer Prusa i3 MK3 (Prusa Research, Prague, Czech Republic) was used. The nozzles were made of hardened steel with diameters of 0.25 mm, 0.4 mm, and 0.5 mm.

### 2.4. Debinding and Sintering

Differential-thermal-analyses and thermogravimetric analyses (DTA/TG) were carried out to study the thermal decomposition/debinding characteristics of the PLA and in order to measure the fraction of ceramics within the filaments. Therefore, a DTA/TG machine Netzsch STA 409 (Erich NETZSCH GmbH & Co. Holding KG, Selb, Germany) was used, the measurements were taken under air atmosphere in the range of 20 °C up to 550 °C with a heating rate of 10 K/min.

Different approaches were investigated for debinding. Two different thermal debinding programs, a simple two-step scheme P1 and a seven-step debinding scheme P2 as shown in Table 3 were applied. The P2 program developed by *Sommer* has a proven track record for debinding of injection molded green bodies which contain a comparable fraction of organics and fine oxide ceramic particles [24]. The water-soluble PEG fraction in the C2 and C3 components was removed prior to thermal debinding by extracting the printed green bodies in a large surplus of distilled water at room temperature for 40 h. The subsequent thermal debinding of the printed scaffolds in air was tried using freestanding components or with components embedded into a powder bed. Powder beds of fine alumina powder APA-05 (Ceralox Alumina, Tucson, AZ, USA) with a medium particle size of 330 nm and coarse alumina powder F100 (IWM Strahltechnik GmbH, Metzingen, Germany) with a medium particle size between 106–150 µm were investigated.

The final sintering of the components that were successfully debindered was performed 1250 °C for 4 h in air atmosphere.

### 2.5. Mechanical Testing

The roundness of the filaments and the distribution of the ceramic particles across the filament cross-section was examined by optical micrographs.

Based on the fact, that only the C2 based components could be successfully debindered and sintered, only such sintered components were. The compressive strength was determined based on the DIN EN ISO 20504 standard for the green bodies and debindered components and according to DIN 51104 standard for the sintered parts. All specimens printed for mechanical testing had a cylindrical shape. The diameter of the green bodies was 10 ± 0.2 mm, the height 10 ± 0.5 mm. The diameter of the sintered C2 cylinders was 4.5 ± 0.1 mm and the height 8 ± 0.2 mm, respectively. The tests were carried out on a universal testing machine Zwick Z100 (Zwick GmbH & Co. KG, Ulm, Germany) at a crosshead speed of 5 mm/min and a preload of 0.5 N.

A mercury porosimeter Pascal 140/440 (CE Instruments, Hindley Green, United Kingdom) was used to analyze the open porosity and the pore size distribution of the sintered cylindrical scaffolds. The pressure range was chosen between 100 kPa and 400 MPa which corresponds to a measurable pore size range of 0.001–100 µm.

## 3. Results

### 3.1. Filament Extrusion

Although the filaments were too brittle to be wound on a spool as originally intended, the extruded rods could be fed to the printer. These rods were 400–500 mm long. Their diameter was 1.75 ± 0.1 mm. This filament diameter is necessary for the uniform printing process, the tolerance is comparable to commercially available, purely thermoplastic filaments.

**Figure 1:**
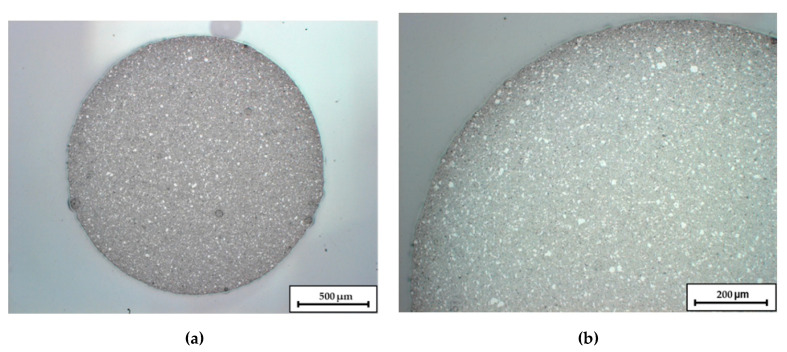
Micrographs of an extruded C1 Filament. Image (**a**) shows the overall cross-section, in image (**b**) the upper left quadrant is enlarged.

Figure 1 shows an optical micrograph image of a filament cross section. Besides the sufficient roundness for the continuous feed into the printer, the images reveal a homogeneous dispersion of the ceramics within the filament.

### 3.2. Rheological Properties

**Figure 2:**
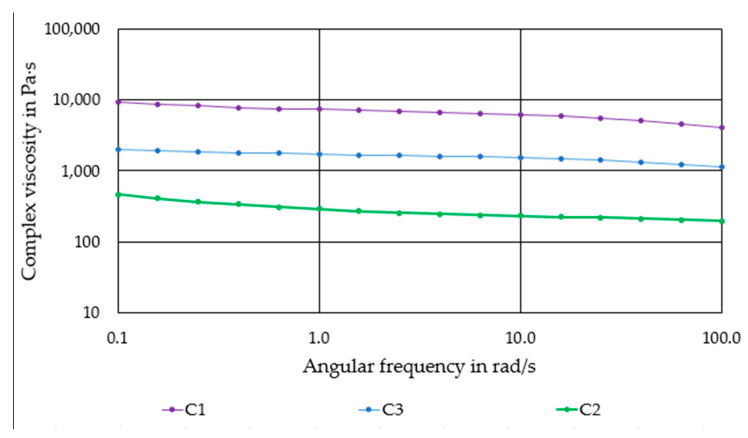
Complex viscosity of the produced compounds as a function of the angular frequency.

Figure 2 shows the flow behavior of the produced compounds. All compounds exhibit a moderate shear thinning behavior. Compound C1 has the highest complex viscosity of ~5000–10,000 Pas. The progressive substitution of PLA by PEG causes a significant reduction of viscosity. Compound C2 has the lowest viscosity of 300–700 Pas.

### 3.3. Printing of the Components

Depending on the geometry to be printed, the nozzle diameter and nozzle temperatures had to be adjusted. The parameters listed in Table 4 were the most suitable for the printing of cylindrical scaffolds. The glass transition point of PLA is 60 °C, this is why the print bed was heated to this value for the pure PLA compound. For the two compounds containing PEG, however, the temperature was reduced to 40 °C, since the PEG was already molten at 50 °C.

**Figure 3:**
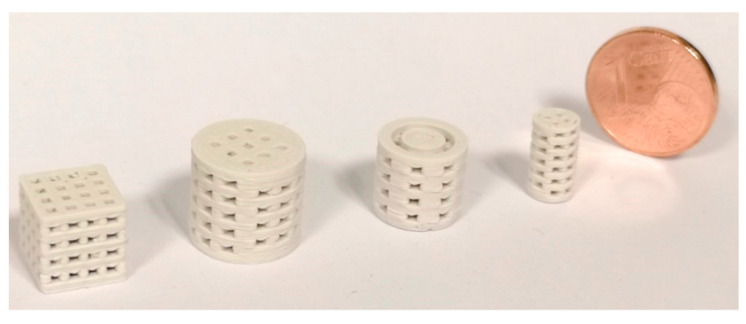
3D printed scaffolds, the nozzle diameter was 0.5 mm, layer height was 0.1 mm.

Printing of the C1 based Filaments required higher nozzle temperatures for smaller nozzle diameters. Otherwise, the viscosity of the material was too high, which led to insufficient flow rates and often to a breaking of the filament at the extruder gears. Figure 3 shows some C1 based scaffolds.

**Figure 4:**
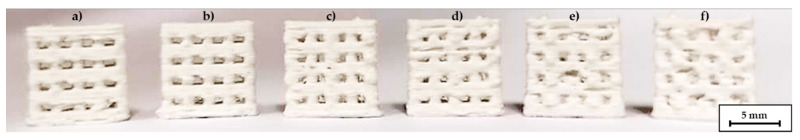
C2 based scaffolds, printed with different temperatures, nozzle diameter 0.4 mm, (**a**) 190 °C, (**b**) 195 °C, (**c**) 200 °C, (**d**) 205 °C, (**e**) 210 °C, (**f**) 215 °C.

**Figure 5:**
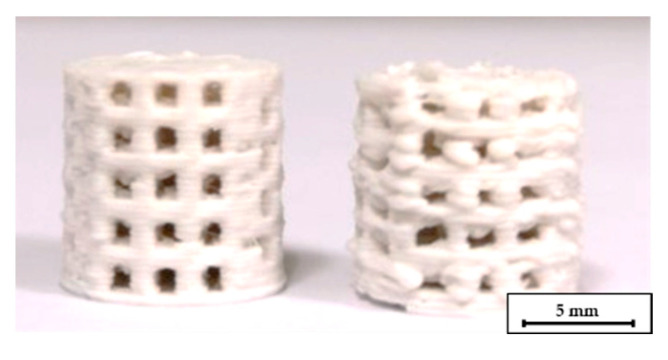
C2 based scaffolds printed at 205 °C, for the left one a 0.4 mm nozzle was used, for the right one a 0.5 mm nozzle.

The addition of high amounts of PEG had a strong influence on the printing quality. Sufficient layer adhesion could not be achieved below 180 °C nozzle temperature. Obviously, at these temperatures, the PLA in the filaments is not heated sufficiently to bond with the underlying layer. Temperatures of 200–205 °C are therefore required for reliable layer adhesion and reproducible printing. However, at 200 °C the filament from compound C2 becomes very low-viscous. As a result, stringing occurred between different features and the level of detail was very low, as shown in Figure 4. The low-viscosity also affected the printing quality with different nozzles. The quality significantly decreased when the scaffolds were printed with a 0.5 mm nozzle instead of a 0.4 mm nozzle at the same temperature, see Figure 5. The increased diameter resulted in an uncontrolled outflow of mass. However, printing at 205 °C was not possible with the 0.25 mm nozzle, therefore the nozzle temperature had to be increased to 215 °C for this nozzle diameter. At this temperature, however, the compound was not printable with the 0.4 mm and 0.5 mm nozzles.

The C3 based filaments contained less PEG than the C2 filaments. For that reason, the printing temperature could be increased by 5 °C to improve the inter layer bonding.

### 3.4. Debinding and Sintering

The C1 based scaffolds were thermally debindered. Without a surrounding powder bed, all components have melted away leading to the destruction of the components. Even embedding into a powder bed of a fine powder APA-05 or a coarse powder F100 did not work satisfactorily. The components did then not melt away anymore, but there was severe distortion and the formation of hollow structures. A successful debinding of the C1 scaffolds was not possible in this study, neither with the P1 program nor with the P2 program.

C2 and C3 were debindered in a two stage debindering process. For the C2 and C3 based specimen a significantly weight loss (of water-soluble PEG) after submerging the components in a water bath for 40 h at room temperature was observed. For the C2 components 90% of PEG could be dissolved and nearly 85% for the C3 components. This is a volume loss of 2 vol.% according to the whole component volume for the C2 based specimens and 15 vol.% for the C3 based specimens, respectively. Both values are in the areas that are postulated in the literature for the creation of pore channels that are necessary for a non-destructive thermal debindering [25].

**Figure 6:**
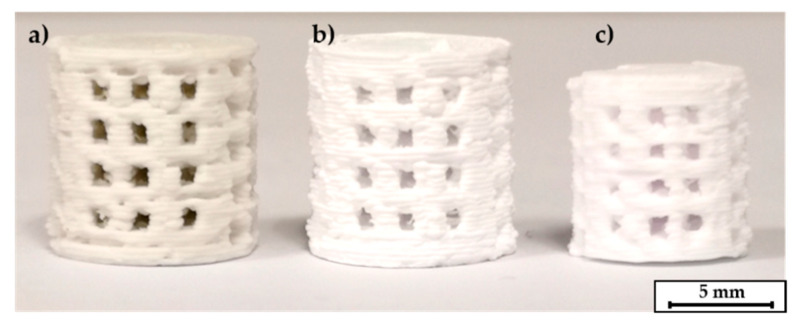
C2 based printed scaffolds: (**a**) green body, (**b**) after debinding, (**c**) as fired.

**Figure 7:**
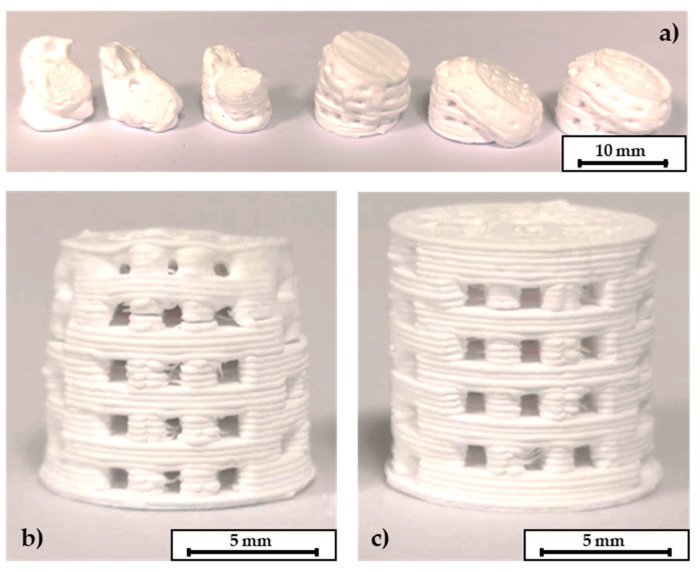
C3 based scaffolds. (**a**) P1 program without a surrounding powder bed leads to destruction of the components. (**b**) P2 program without a powder bed and (**c**) debinding within a powder bed. The layer thickness of these scaffolds was 0.25 mm, height of the scaffolds 10 mm, pore size 1 mm × 1 mm.

The following step of thermal debinding did not require a surrounding powder bed for the C2 based components. Both debinding strategies P1 and P2 were applicable. The open-pore network that was created after the dissolution of the PEG seems to be permeable enough to release the volatile components of the thermal debindering process. The specimens could withstand the thermal expansion of the residual PLA binder. A printed, a debindered, and a sintered C2 based scaffold are shown in Figure 6. The components retain their shape during the debindering and show a homogeneous shrinkage during final sintering. In contrast to this the C3 specimens could only be debindered in a dimensionally stable manner with the P2 program and the aid of the fine APA-05 powder. When the P1 program was used, the components did not keep their dimensions, see Figure 7a. This effect could be reduced by using a powder bed, but not completely eliminated. However, it was possible to debinder C3 based scaffolds successfully when the P2 program was used. Without a surrounding powder bed the dimensional accuracy was quite low, see Figure 7b. Better results were obtained when the debinding took place in an APA-05 powder bed, see Figure 7c. Using the coarse F100 powder was not effective here either.

**Figure 8:**
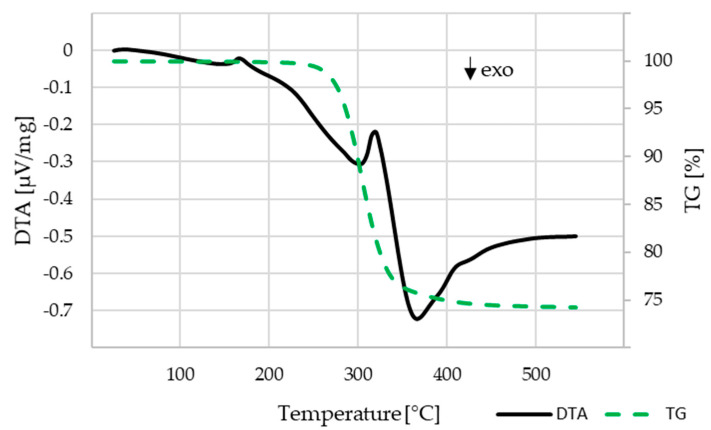
DTA-TG analysis for the C1 compound, heating rate 10 K/min, air atmosphere.

The investigation on the binder decomposition behavior was carried out for the C1 components that contained the highest amount of PLA, which had to be thermally burnt out. PLA is the only binder component for C1 and after water debindering makes up for the majority of the residual binder in C2 and C3. The results are shown in Figure 8. The mass loss starts at about 230 °C and ends at about 450 °C. With the remaining mass of 74.2 wt.% a volume content of 54 vol.% β-TCP within the component can be calculated, which is close to the theoretical value of 55 vol.%.

### 3.5. Mechanical Characterization

**Figure 9:**
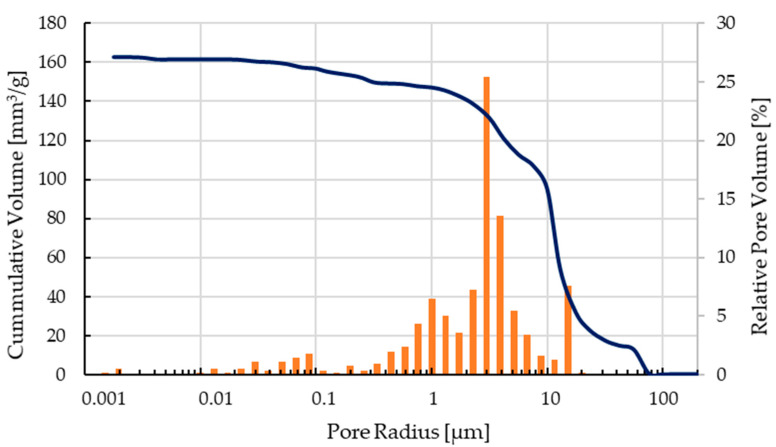
Pore size distribution of a sintered C2 based scaffold.

The mechanical characterization focused on C2 components as only these scaffolds could be printed, debindered, and sintered without difficulty and showed the best results after optical inspection. The pore size distribution of a sintered scaffold as shown in Figure 6 can be seen in Figure 9. The open porosity measured by the mercury porosimeter is about 35%. Most of the micropores are in the range between 0.4–12 µm, the calculated medium pore size was 9.8 µm.

**Figure 10:**
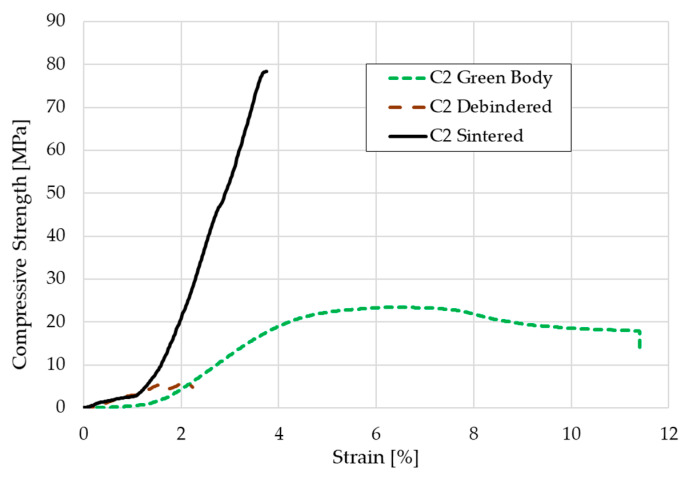
Stress-strain-curve for a C2 based green body, brown body, and sintered scaffold.

The compressive strength was calculated by testing five green bodies, five debindered components and five sintered cylindrical scaffolds according to the mentioned standard. The stress-strain-curve for one representative of each group each is shown in Figure 10. The medium compressive strength was 26.9 ± 4.1 MPa for the green bodies, 5.2 ± 0.5 MPa for the debindered bodies, and 72.4 ± 12.4 MPa for the sintered scaffolds.

**Figure 11:**
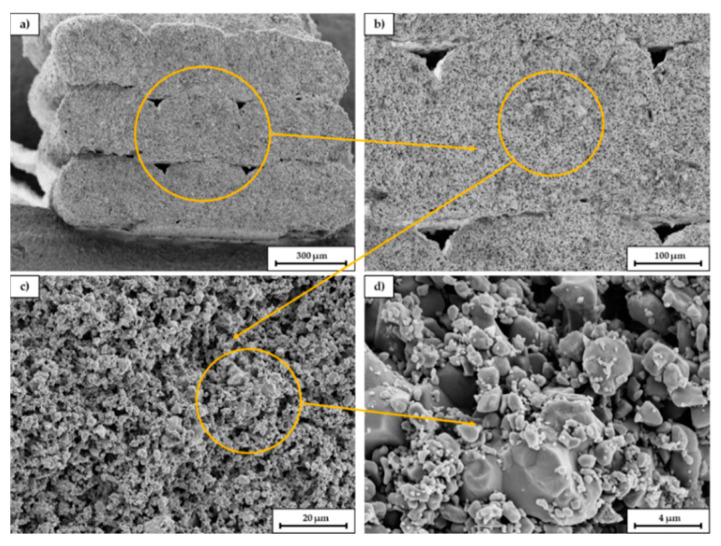
SEM images of a fracture surface of a sintered C2 based scaffold. The magnification increases from (**a**–**d**), the orange circle is the enlarged area.

Figure 11 shows SEM images of a broken sample at different magnifications. The fracture surface is oriented parallel to the longitudinal axis of the scaffold and thus orthogonal to the printed layers. In picture (a) the single extruded struts can be seen. The gaps between the single struts (within the orange circle) indicate that the layer bonding between the single layers is not perfect, probably due to the high viscosity of the extruded paste. As already explained in 3.3 it was necessary to lower the nozzle temperature to 200 °C due to the presence of low melting PEG, however for printing of PLA the viscosity of the compound was probably slightly too high. Most commercially available PLA filaments are printed between 210–215 °C. Picture (b) and (c) indicate the relatively high porosity of the scaffold and the homogeneous pore size distribution. In picture (d) sinter necks between the primary particles can be identified as well as a relatively high amount of fine particles. The amount of fracture surfaces is surprisingly low.

## 4. Discussion

The results of the rheological investigations have shown that the viscosity of the compounds is in a range that is well suited for processing by FDC. This observation is confirmed as compound preparation and further processing into filaments could be performed without difficulty. The lower viscosity of compounds C2 and C3 compared to C1 suggests that an increase in the ceramic content is definitely feasible for C2 and C3. However, an increase in the ceramic content for C2 and C3 would result in more brittle filaments. Furthermore, a reduction in the proportion of polymer in the overall system may lead to reduced adhesion of the printed layers. During debinding, a higher filler content will ensure that the printed components can be debindered more easily and that a lower shrinkage and deformation can be achieved. Due to the lower inter particle distance in the green body sinterability will probably be promoted. The use of a more ductile polymer blend as matrix material is recommended for future work. This makes it easier to handle and coil up the filaments. When selecting the material, the printability and medical requirements must be taken into account.

The quality of the printed parts, especially the C2 and C3 based components, differed strongly with the nozzle temperature. The glass transition temperature of the used PLA was higher than the melting point of the PEG. This means, that the low viscosity of the PEG at higher temperatures decreased the accuracy of the shaping process. On the other hand, decreasing the temperature led to a higher accuracy, but also to an increased risk of layer delamination caused by a lower inter-layer bonding. Another approach to fix this issue might be the addition of tackifiers during compounding. This could make it possible to lower the nozzle temperature while increasing the layer adhesion. An additional option to increase the flexibility of the filaments is to add plasticizers. This might lower the risk of breaking the filament during the feeding into to extruder or for retraction movements. On top of that the filaments can be spooled, increasing the length of available filament for one printing process without loading a new one, which also influences the printing quality. The influence of tackifiers and plasticizers on the printing quality and the debinding behavior are to be studied in detail in upcoming investigations.

The shaping of complex scaffolds via FDC was possible for every component used. The general tendencies are: the higher the amount of PLA and the lower the PEG content, the higher the printing quality and the easier the printing. On the other hand, a successful debinding of components that only contained PLA was not possible within this study. Either the components started to flow when no powder bed was used or the ceramic powders migrated to the surface of the component when a powder bed was used, leading to voids in the inner area of the scaffolds and cylinders. For a successful debinding a high amount of bioceramics within the rods/filaments as well as a soluble organic component next to the PLA was necessary to allow a two-step debinding process. The C2 based components contained the highest amount of PEG, the resulting pore network was sufficient for the following step of thermal debinding. Of course, a higher amount of PEG makes the thermal debinding easier. On the other hand, it leads to a lower printing quality and, when a critical value of PEG is reached, it definitely will lead to the destruction of the printed components during dissolving. Therefore, choosing the proper amount of PEG within the organic backbone is essential for the FDC process.

Except for the high strain values, the curves in the stress-strain diagram show the typical fracture behavior of ceramics and green bodies. The sintered cylinders in particular only have a linear-elastic stress curve. With the green bodies, the elongation at break is of course more pronounced due to the mobility of the polymer chains. The elongation of nearly 12% might be realistic for the green parts, but a deformation of more than 2% for the debindered and nearly 4% for the sintered cylinders is not realistic. This relatively high offset is presumably due to inaccuracies in the displacement transducer on the machine used. Nevertheless, the compressive strength of the sintered cylinders seems realistic. In previous studies of the authors other additive manufacturing approaches for the shaping of β-TCP cylinders were carried out. Cylinders made by binder jetting technique showed a medium compressive strength of about 4.4–5.6 MPa. When the indirect Solid Freeform Fabrication approach was used—in this case a ceramic slurry was casted into a sacrificial thermoplastic mold made via FFF—the compressive strength that was reached was nearly 22 MPa [26]. We assume, that the relatively high amount of β-TCP with more than 54 vol.% within the component compared to the other two approaches as well as many other AM techniques decreases the porosity of the debindered part. Additionally, the consolidation of the 1.75 mm filaments in the 0.4 or 0.5 mm nozzle leads to a relatively compact ceramic primary particle structure within the green body. The particles have a lot of contact zones that improve the diffusion process during sintering which leads to a higher consolidation. The denser particle packing increases the strength. However, the highest possible porosity should be aimed for cancellous bones, which, depending on the author, should be between 50–90% [15,16]. In this respect, the compaction of the scaffolds made by FDC is negative, because the open porosity was only 35%. Furthermore, the compressive strength is far above the usual values for cancellous bone of 2–20 MPa. With regard to bone remodeling, these structures would therefore not be suitable for cancellous bone replacement, but possibly for load-bearing applications.

Recent studies showed that the combination of macro- and micropores within a bioceramic scaffold improve the ingrowth of bone cells into the implant. From this point of view, the test specimens produced using FDC are interesting. With this manufacturing variant, the macroporosity can be influenced by CAD modelling and the choice of the printer nozzle. The resulting micropores in the range of 0.4–12 µm result from the compounding measures and probably also from the debinding and sintering strategy.

The direct comparison of different additive manufacturing processes is always difficult and depends on the materials to be processed and the desired component properties. Depending on which parameter you focus on, the different manufacturing processes offer advantages and disadvantages. A direct comparison is therefore often subjective. In the following, however, a brief attempt will be made to compare the β-TCP scaffolds printed using FDC with other, widely used additive manufacturing processes.

With powder-based processes such as selective laser sintering (SLS) or binder jetting, there are always increased requirements for occupational safety with regard to dust. The filaments processed in this study can be processed like commercially available filaments made of pure PLA without great effort. It is not necessary to evacuate or fill the building chamber with inert gas, as is the case with the SLS process. When excess powder is recycled, there is always the risk of segregation and thus a change in the properties of the powder and, ultimately, of the component. This is not the case with homogeneously prepared filaments. In the case of stereolithographic processes (such as SLA, DLP, LCM, etc.), the health risk from the resins is also higher than in the FDC process. Post-processing after printing is minimal, especially for components that do not require support structures. In the case of the competing processes mentioned above, the removal of excess powder or uncrosslinked resin is absolutely necessary, which is associated with the risk of component damage. Furthermore, the printing speed and the material throughput in the FDC process are relatively high, especially compared to stereolithographic processes. In comparison to components that were printed using binder jetting, handling is much better due to the significantly higher strength of the green body. Basically, FDC printers are widespread and much cheaper than other additive manufacturing processes.

A disadvantage to be mentioned is that the achievable resolution and thus the level of detail with the FDC process is usually significantly lower than with SLS, binder jetting and, above all, the stereolithographic process. Standardized scaffold geometries like cylinders can be easily produced using FDC, but imaging of the filigree cancellous bone is almost impossible. If overhangs and hollow structures are printed, support structures are absolutely necessary. These must either be water-soluble or mechanically removed, which might be a complex process. With powder-based processes, the component is supported by surrounding powder and no additional support structures are needed. Successful debinding requires, among other things, a high proportion of ceramic in the filament, which leads to the filament becoming brittle. This makes it much more difficult to process them, which can lead to errors in the printing process.

As already mentioned, a generally valid comparison between different additive manufacturing processes is difficult. In summary, however, it can be said that the FDC process offers great potential for printing bioceramic materials or ceramic–plastic composites quickly and in sufficient quality so that these components can be used for medical applications.

## Figures and Tables

**Table 1:** Composition of the three different compounds C1, C2, and C3.

Compound	β-TCP (vol.%)	PLA (vol.%)	PEG (vol.%)
C1	55	45	0
C2	55	18	27
C3	55	27	18

**Table 2:** Temperature zones in the single screw extruder during filament extrusion.

Compound	Zone	1	2	3	4	5
C1	**Temperature (°C)**	160	190	200	205	200
C2, C3		160	180	185	190	185

**Table 3:** Debinding strategy for the different compounds.

	Step	1	2	3	4	5	6	7
	**Heating rate (K/h)**	30	60	-	-	-	-	-
**P1**	**Temperature (°C)**	500	900	-	-	-	-	-
	**Dwell (h)**	3	3	-	-	-	-	-
	**Heating rate (K/h)**	68	2	9	5	9	14	200
**P2**	**Temperature (°C)**	168	198	250	282	350	420	800
	**Dwell (h)**	0	0	0	0	0	0	3

**Table 4:** Printing parameters for the different compounds.

Printer Parameter	C1			C2		C3	
**Nozzle Diameter (mm)**	0.25	0.4	0.5	0.4	0.5	0.4	0.5
**Temperature Nozzle (°C)**	230	220	215	200	200	205	205
**Temperature Heatbed (°C)**	60	60	60	40	40	40	40

## Data Availability

The data presented in this study are available on request from the corresponding author.
